# Different transporter systems regulate extracellular GABA from vesicular and non-vesicular sources

**DOI:** 10.3389/fncel.2013.00023

**Published:** 2013-03-13

**Authors:** Inseon Song, Kirill Volynski, Tanja Brenner, Yuri Ushkaryov, Matthew Walker, Alexey Semyanov

**Affiliations:** ^1^RIKEN Brain Science InstituteWako-shi, Saitama, Japan; ^2^Department of Clinical and Experimental Epilepsy, UCL Institute of NeurologyLondon, UK; ^3^Medway School of Pharmacy, University of KentChatham Maritime, UK; ^4^Department of Neurodynamics and Neurobiology, University of Nizhny NovgorodNizhny Novgorod, Russia

**Keywords:** GABA transporter, tonic GABA_A_ conductance, mutant α-latrotoxin, GABA source, extrasynaptic signaling, GABA, interneurons

## Abstract

Tonic GABA type A (GABA_A_) conductance is a key factor regulating neuronal excitability and computation in neuronal networks. The magnitude of the tonic GABA_A_ conductance depends on the concentration of ambient GABA originating from vesicular and non-vesicular sources and is tightly regulated by GABA uptake. Here we show that the transport system regulating ambient GABA responsible for tonic GABA_A_ conductances in hippocampal CA1 interneurons depends on its source. In mice, GABA from vesicular sources is regulated by mouse GABA transporter 1 (mGAT1), while that from non-vesicular sources by mouse GABA transporters 3/4 (mGAT3/4). This finding suggests that the two transporter systems do not just provide backup for each other, but regulate distinct signaling pathways. This allows individual tuning of the two signaling systems and indicates that drugs designed to act at specific transporters will have distinct therapeutic actions.

## Introduction

Tonic activation of extrasynaptic GABA type A receptors (GABA_A_) by ambient GABA is a major form of GABAergic signaling in the central nervous system (Semyanov et al., 2004; Farrant and Nusser, 2005; Glykys and Mody, 2007a; Walker and Semyanov, 2008). It can be recorded in voltage-clamped neurons as the part of holding current (*I*_hold_) that is sensitive to GABA_A_ receptor antagonists (termed tonic GABA_A_ current). The expression of tonic GABA_A_ currents is cell-type specific, and this specificity determines the network effect of this form of signaling (Semyanov et al., 2004). For example, in the hippocampal CA1 region, tonic GABA_A_ currents are significantly larger in interneurons than in pyramidal cells (Semyanov et al., 2003; Scimemi et al., 2005), and this difference may be due to a more efficient GABA uptake around pyramidal cells. Tonic currents also exert different effects on interneuron and pyramidal cell excitability. In the adult brain, GABA hyperpolarizes hippocampal pyramidal neurons (Glickfeld et al., 2009), whereas it depolarizes interneurons (Michelson and Wong, 1991; Banke and McBain, 2006; Vida et al., 2006; Song et al., 2011). Consequently, tonic GABA_A_ conductances have inhibitory effects on pyramidal neurons, whereas low tonic GABA_A_ conductances have excitatory effects on interneurons (Song et al., 2011). When the ambient GABA concentrations increase, tonic GABA_A_ conductances become larger and the shunting overpowers the excitatory effect of depolarization, rendering the overall effect of the tonic GABA_A_ conductance inhibitory. This phenomenon is particularly interesting given the diverse roles of interneurons in the hippocampus, such as rhythmic activity, synchronization of pyramidal cells firing, feedforward, and feedback inhibition (see for review, Kullmann, 2011). Thus mechanisms changing ambient GABA concentrations around interneurons, such as GABA release and uptake, can have profound functional effects. Ambient GABA originates from a number of synaptic and non-synaptic sources in the brain. The magnitude of tonic GABA_A_ currents in rat CA1 pyramidal neurons correlates with the frequency of spontaneous inhibitory post-synaptic currents (sIPSCs) when rat GAT1 (rGAT1) is blocked, suggesting a synaptic contribution to ambient GABA around these cells (Glykys and Mody, 2007b). Astrocytic GABA release has been demonstrated in the olfactory bulb (Kozlov et al., 2006) and cerebellum (Lee et al., 2010). Dendritic exocytotic GABA release has been reported in the neocortex (Zilberter et al., 1999). Extracellular GABA is cleared by GABA transporters, classified as mouse GABA transporter 1 (mGAT1), mGAT2, mGAT3, and mGAT4 in the mouse [homologs in rat: mGAT1 ~ rGAT1, mGAT2 ~ betaine/GABA transporter 1 (BTG1), mGAT3 ~ rGAT2, mGAT4 ~ rGAT3]. In rats, rGAT1 and rGAT2/3 blockers synergistically modulate tonic GABA_A_ conductances (Keros and Hablitz, 2005; Jin et al., 2011). The relative impact of different transporters on ambient GABA originating from different sources, however, has been unknown. Here we show in mouse hippocampal CA1 *stratum (str.) radiatum* interneurons that mGAT1 and mGAT3/4 selectively regulate tonic GABA_A_ currents mediated by GABA originating from vesicular and non-vesicular sources, respectively.

## Materials and methods

### Ethical approval

All procedures involving animals were approved by the institutional Animal Care and Use committee at RIKEN.

### Slice preparation

Hippocampal slices (350 μm) were obtained from 3- to 4-week-old C57BL/6J mice. After dissection, the hippocampi were sliced using a vibration microtome (Microm HM650V, Germany). Transverse hippocampal slices were prepared in ice-cold slicing solution containing (in mM): 87 NaCl, 2.5 KCl, 7 MgCl_2_, 0.5 CaCl_2_, 26.2 NaHCO_3_, 1.25 NaH_2_PO_4_, 25 glucose, and 50 sucrose, and saturated with 95% CO_2_/5% O_2_. After preparation, the slices were maintained at room temperature in a submerged chamber with storage solution containing (in mM): 119 NaCl, 2.5 KCl, 1.3 MgSO_4_, 1 CaCl_2_, 26.2 NaHCO_3_, 1 NaH_2_PO_4_, and 11 glucose, and saturated with 95% CO_2_/5% O_2_.

### Whole-cell patch clamp recording

After 1 h incubation, slices were transferred to the recording chamber and superfused at 32–34°C with external solution (same as above, but containing 2.5 mM CaCl_2_). AMPA/kainate, NMDA, and GABA_B_ receptors were blocked with 25 μM NBQX, 50 μM APV, and 5 μM CGP52432 (Tocris Cookson, Bristol, UK), respectively.

α-Latrotoxin from the black widow spider venom stimulates neurotransmitter release by acting on presynaptic receptors (Ushkaryov et al., 2008; Silva et al., 2009). α-Latrotoxin mutant (LTX^N4C^) in which four amino acids were inserted between the main domains (Ichtchenko et al., 1998) was used to stimulate spontaneous vesicular exocytosis (Volynski et al., 2003). Stock aliquots of LTX^N4C^ (17–42 nM) were stored at −28°C in non-freon Nihon freezer GS-1356HC (Japan). LTX^N4C^ was focally applied to the recording chamber at a concentration of 0.1 nM near the recording pipette after stopping the superfusion (Capogna et al., 2003). Perfusion was resumed when the frequency of sIPSCs began to increase (0.5–10 min after toxin application). Such an interruption in perfusion had no effect on control recordings. In experiments where vesicular release was blocked, slices were pre-treated for at least 2.5 h in 4 μM bafilomycin A1 (Wako Chemicals, Japan) while control slices from the same animal were kept in external solution without bafilomycin A1. All recordings were made from CA1 *str.radiatum* interneurons visually identified with an infrared differential interference contrast microscope (Olympus BX51WI, Japan). Whole-cell pipettes used in voltage-clamp recordings contained (in mM): 130 CsCl, 8 NaCl, 10 Cs-HEPES, 2 EGTA, 0.2 MaCl_2_, 2 MgATP, 0.3 Na_3_GTP, and 5 QX314Br (pH 7.2, osmolarity 295 mOsm). The data were acquired with a Multiclamp700B amplifier (Molecular Devices, Sunnyvale, CA), filtered at 3 kHz, and digitized at 10 kHz using a NI PCI-6221 data acquisition card (National Instruments, Austin, TX). The data were analyzed without further re-sampling. Sample traces were also taken at the same rate; the long traces (>30 s) showing the *I*_hold_ were re-sampled at 10 Hz for illustration purposes. Frequency, amplitude, and area of sIPSCs were analyzed off-line with MiniAnalysis (Synaptosoft Inc, Decatur, GA) and Clampfit (Molecular Devices) programs.

Whole-cell voltage-clamp recordings were performed at −70 mV. Tonic GABA_A_ currents (Δ*I*_hold_) were calculated as the difference between the baseline *I*_hold_ and the *I*_hold_ in the presence of the drug affecting tonic GABA_A_ currents. To estimate *I*_hold_ in control and drug conditions, we plotted all-points histograms over 20 s of recordings in each case (Glykys and Mody, 2007b). These histograms have a peak which corresponds to mean *I*_hold_ while fluctuations in the holding current affect the width and skewness of the histograms. This method was specifically developed to overcome bias due to the presence of IPSCs on the *I*_hold_ measurements. The time-averaged currents mediated by sIPSCs (*I*_spont_) were calculated as the mean charge transfer of sIPSCs (area under the IPSCs) multiplied by their frequency (Semyanov et al., 2003).

### Statistics

Statistical analysis was performed using Excel (Microsoft, USA) and Origin8 (OriginLab, USA). Data were presented as ± S.E.M; ^*^P < 0.05, ^**^P < 0.01 paired, unpaired, or one-sample Student's *t*-test as stated in the text. A *P*-value of less than 0.05 was considered statistically significant. Two-Way ANOVA was used to assess the effects and interaction of GABA transporter blockers and bafilomycin A1.

## Results

### Tonic GABA_A_ conductance is regulated by both mGAT1 and mGAT3/4 transporters

**Figure 1:**
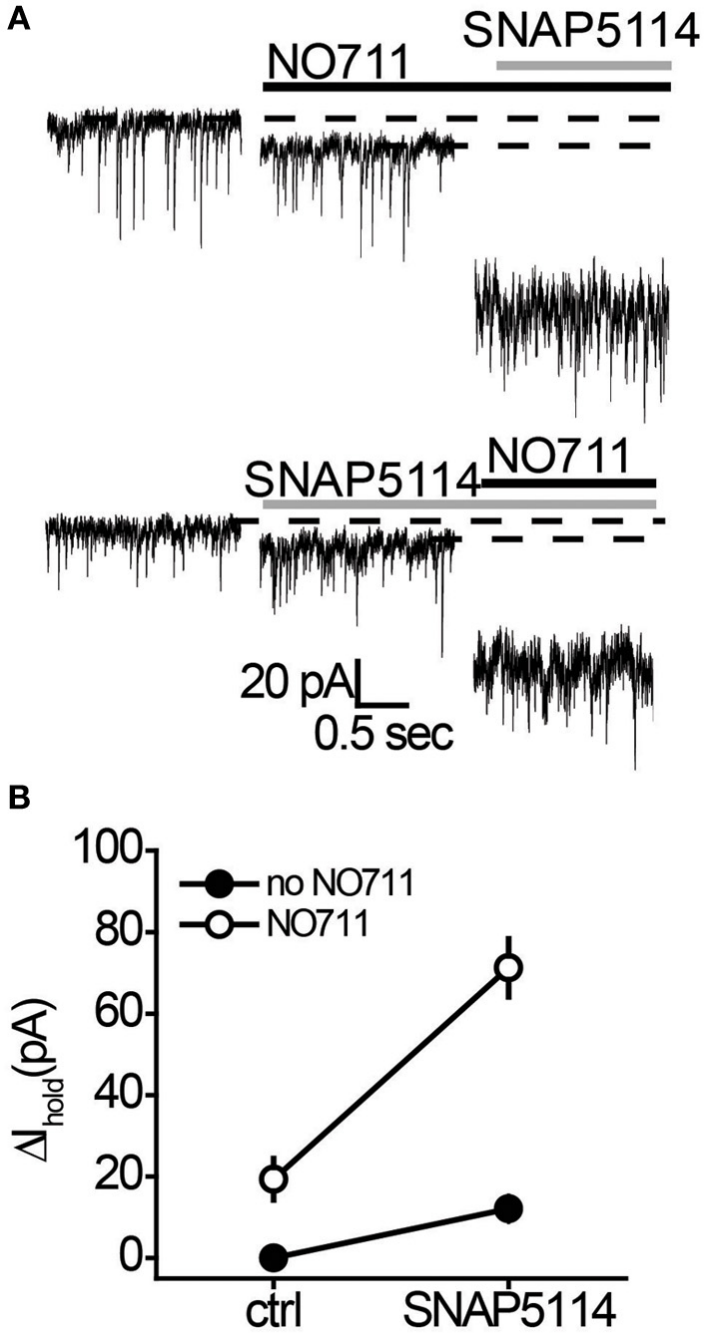
**NO711 and SNAP5114 have a synergistic effect on the tonic GABA_A_ current. (A)** Changes in *I*_hold_ produced by SNAP5114 following NO711 application (*upper traces*) and by NO711 following SNAP5114 application (*lower traces*). *Dashed lines*—baseline or drug-blocked levels of *I*_hold_; *black line*—application of NO711, *gray line*—application of SNAP5114. **(B)** Summary graph of changes in *I*_hold_ produced by separate applications and co-application of SNAP5114 and NO711, showing a strong interaction between the two transporter antagonists (*n* = 6; *F*_(1, 5)_ = 18.72, *P* < 0.01, Two-Way ANOVA).

**Figure 2:**
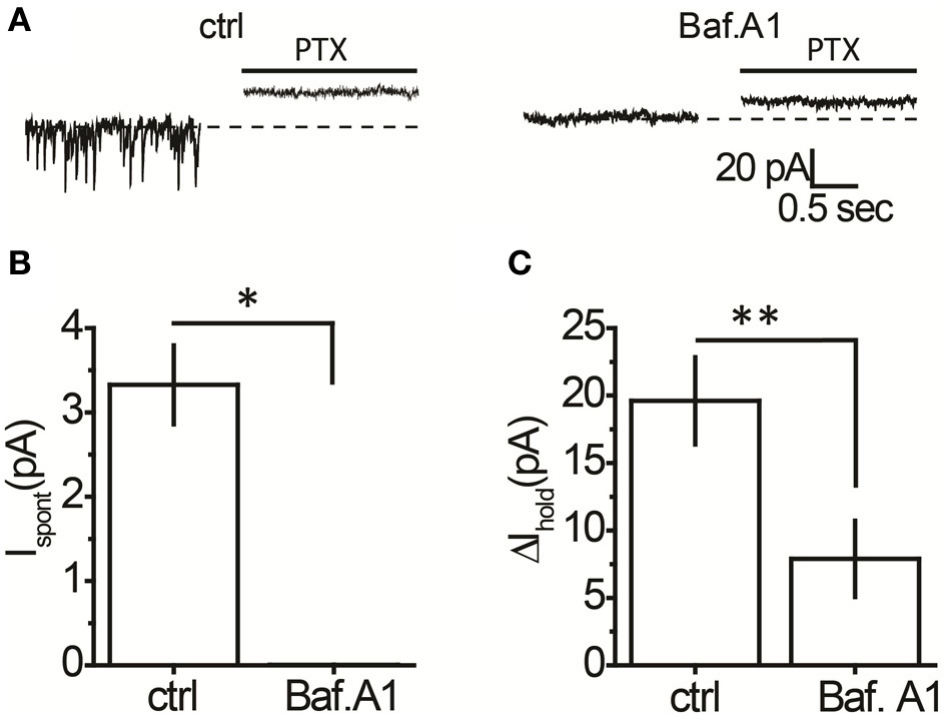
**A proportion of the tonic GABA_A_ current in hippocampal interneurons is independent of vesicular GABA release. (A)** Representative traces showing picrotoxin (PTX)-sensitive tonic current in a control slice and a slice treated with bafilomycin A1 (*Baf. A1); black line—*PTX application. **(B)** Mean *I*_spont_ in control (*n* = 14) and bafilomycin A1-treated slices (*n* = 6). **(C)** Mean tonic GABA_A_ current (Δ*I*_hold_, *n* = 14) in control and bafilomycin A1-treated slices (*n* = 6). ^*^P < 0.05, ^**^P < 0.01, unpaired *t*-test.

sIPSCs and *I*_hold_ were recorded in voltage-clamped CA1 *str.radiatum* interneurons in mouse hippocampal slices in the presence of α-amino-3-hydroxy-5-methyl-4-isoxazolepropionic acid (AMPA)/kainate, N-methyl-D-aspartate (NMDA), and GABA type B (GABA_B_) receptor antagonists to isolate GABA_A_ receptor currents. The transient inward currents were mediated by GABA_A_ receptors (sIPSCs) and blocked by application of the GABA_A_ receptor antagonist picrotoxin (100 μM; Figure 2A). Picrotoxin also produced a shift in *I*_hold_, which represents the tonic GABA_A_ current. Then we tested how different transporter subtypes regulate this baseline tonic GABA_A_ current. The mGAT1 blocker NO711 (10 μM) and the mGAT3/4 blocker SNAP5114 (100 μM) both significantly increased *I*_hold_ (Table 1, Figure 1), suggesting that elimination of either transporter subtype leads to increases in ambient GABA. NO711 and SNAP5114 together increased *I*_hold_ more than the sum of the effects of individual blockers suggesting a synergetic effect of the two drugs on extracellular GABA elevation.

**Table 1:** **Effect of mGAT1 and mGAT3/4 blockers on *I*_hold_ in mouse interneurons**.

	**Δ/_hold_ (NO711)**	**Δ/_hold_ (SNAP5114)**	***P***_**(NO711 vs.SNAP5114)**_
Control	19.35 ± 5.09 (*n* = 6)	12.12 ± 2.28 (*n* = 7)	0.100
Bafilomycin A1	7.56 ± 1.74 (*n* = 6)	17.03 ± 4.02 (*n* = 6)	0.028^*^
*P*_(control vs. baf. A1)_	0.026^*^	0.149	

Data are presented as mean ± S.E.M; P-values for unpaired t-test,

* *P* < *0.05*.

### Tonic GABA_A_ conductance is mediated by GABA released from both vesicular and non-vesicular sources

**Figure 3:**
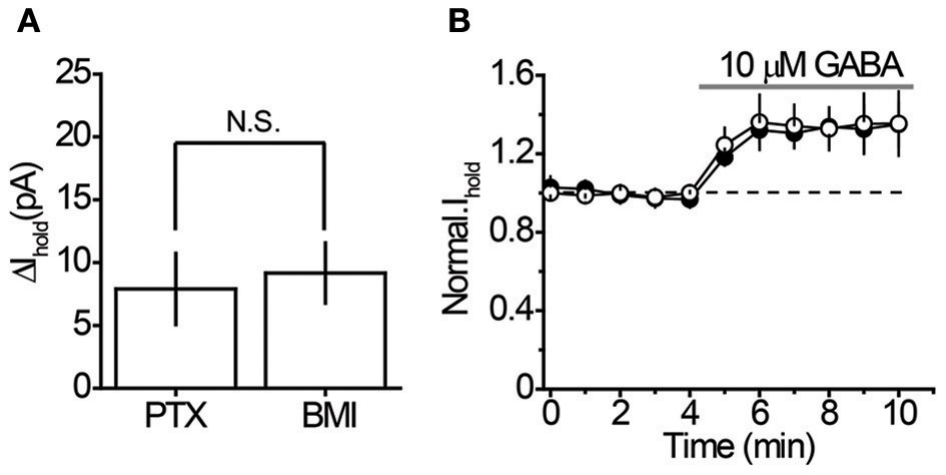
**Characteristics of the tonic GABA_A_ current in bafilomycin A1-treated slices. (A)** 100 μM picrotoxin (PTX, *n* = 6) and 25 μM bicuculline (BMI, *n* = 5) revealed similar tonic GABA_A_ current in CA1 interneurons of bafilomycin A1-treated slices. **(B)** Sensitivity of *I*_hold_ to exogenous GABA (10 μM) in control (*n* = 6) and bafilomycin A1-treated slices (*n* = 7). *Black circles*—*I*_hold_ in control slices, *white circles*—*I*_hold_ in bafilomycin A1-treated slices normalized to corresponding baseline values (dashed line). *Gray line*—GABA application. N.S. *P* > 0.05, unpaired *t*-test.

It was unclear whether GABA elevations during blockade of each transporter system originated from the same or different sources. Indeed, the different locations of rGAT1 (which is predominantly located on neurons) and rGAT3 (which is located on astrocytes) indicate that these transporters may specifically regulate GABA originating from different sources (Ribak et al., 1996; Heja et al., 2009, 2012; Shigetomi et al., 2012). First, we identified the proportion of tonic GABA_A_ conductance in interneurons that is mediated by non-vesicular GABA release. We compared the tonic GABA_A_ current in control slices and in slices pretreated with a selective inhibitor of vacuolar H^+^-ATPases, bafilomycin A1, which prevents GABA loading into synaptic vesicles and thus vesicular GABA release. The slices pretreated with bafilomycin A1 were completely devoid of sIPSCs, confirming total blockade of vesicular release (Figures 2A,B). In these slices, application of picrotoxin revealed significant tonic GABA_A_ currents in CA1 interneurons (7.9 ± 2.8 pA, *n* = 6, *P* = 0.028 one-sample *t*-test), which constituted 40% of the tonic GABA_A_ current revealed by picrotoxin in control slices not treated with bafilomycin A1 (19.6 ± 3.2 pA, *n* = 14, *P* = 0.015 one-sample *t*-test; Figure 2C; *P* = 0.0031 for the difference between control and bafilomycin A1-treated slices, unpaired *t*-test). Because picrotoxin also blocks homomeric glycine receptors, we confirmed that tonic current in bafilomycin A1-treated slices is GABAergic using 25 μM bicuculline (9.17 ± 2.40 pA, *n* = 6, *P* = 0.007 one-sample *t*-test; Figure 3A). Because bafilomycin A1 also affects exocytosis and therefore can potentially affect the neuronal receptor density (Johnson et al., 1993; Presley et al., 1997), we tested whether the responses to exogenous GABA were affected by bafilomycin A1 treatment. The tonic current produced by exogenous application of 10 μM GABA did not differ significantly between control and treated slices (Figure 3B).

### mGAT3/4 regulates GABA originating from non-vesicular sources

**Figure 4:**
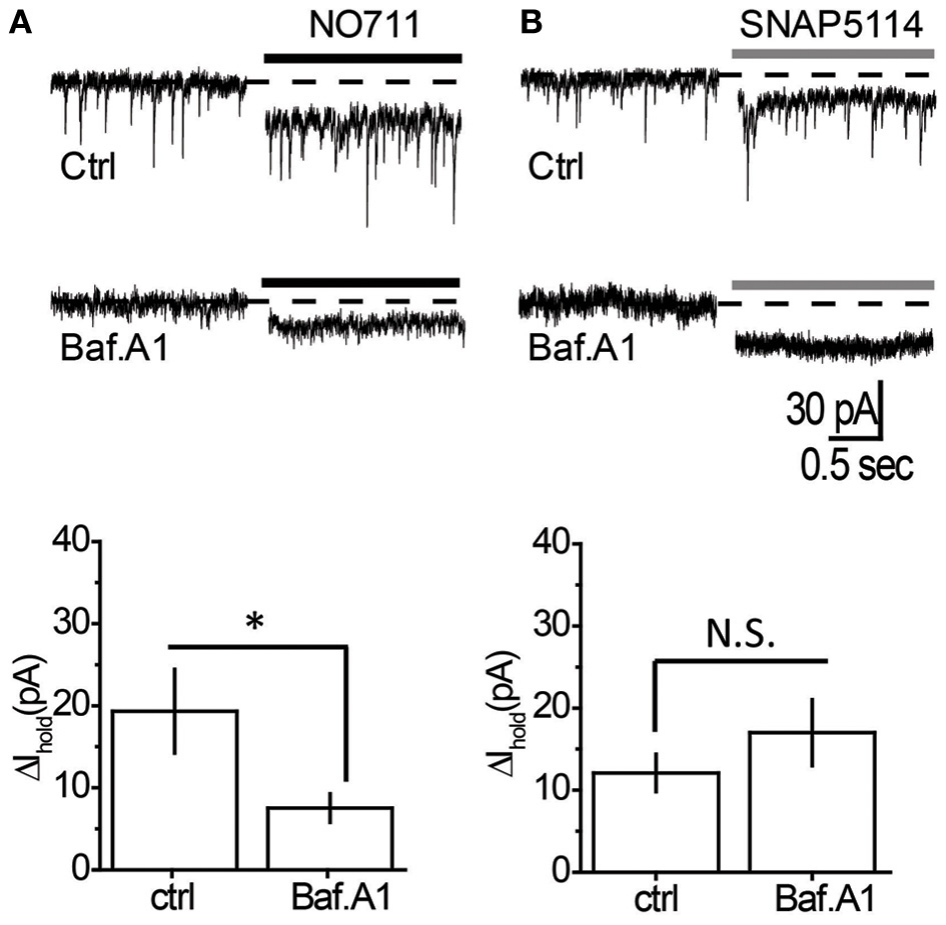
**Tonic GABA_A_ current mediated by non-vesicular GABA release is regulated by mGAT3/4, but not by mGAT1. (A)** *Top*: Changes in *I*_hold_ produced by NO711 in control (*upper trace*) or bafilomycin A1-treated slices (*lower trace*). *Dashed line*—baseline level of *I*_hold_, *black line*—application of NO711. *Bottom*: Summary graph showing changes in *I*_hold_ produced by NO711 in control (ctrl, *n* = 6) and bafilomycin A1-treated (Baf. A1, *n* = 6) slices. **(B)** *Top*: Changes in *I*_hold_ produced by SNAP5114 in control (*upper trace*) or bafilomycin A1-treated slices (*lower trace*). *Dashed line*—baseline level of *I*_hold_, *gray line*—application of SNAP5114. *Bottom*: Summary graph showing changes in *I*_hold_ produced by SNAP5114 in control (ctrl, *n* = 7) and bafilomycin A1-treated (Baf. A1, *n* = 6) slices. ^*^P < 0.05; N.S. *P* > 0.05, unpaired *t*-test.

We then tested the effect of mGAT1 and mGAT3/4 blockers on the tonic GABA_A_ current in the presence of bafilomycin A1. The mGAT1 blocker NO711 led to a smaller increase in the tonic GABA_A_ current in the absence of vesicular release (Table 1, Figure 4A). However, the increase was significant (*P* = 0.007, paired *t*-test), suggesting that mGAT1 limits the contribution of both synaptic spillover and also non-vesicular release to ambient GABA concentrations. In contrast, the tonic GABA_A_ current increase caused by mGAT3/4 blocker SNAP5114 was not significantly different in the absence or presence of vesicular release (Table 1, Figure 4B), suggesting that, in slices at baseline conditions, mGAT3/4 regulates GABA originating largely from non-vesicular sources. We then asked whether the effect of bafilomycin A1 depended on which type of GABA transporters was blocked. Using a Two-Way ANOVA we found that the interaction between bafilomycin A1 and uptake inhibitor is significant [*F*_(1, 21)_ = 434, *p* = 0.025], indicating that bafilomycin A1 has a significantly greater effect in the presence of a mGAT1 blocker than mGAT3/4 blocker and suggesting also that mGAT1 mainly controls the uptake of GABA released by exocytosis.

### mGAT1 regulates GABA originating from vesicular sources

**Figure 5:**
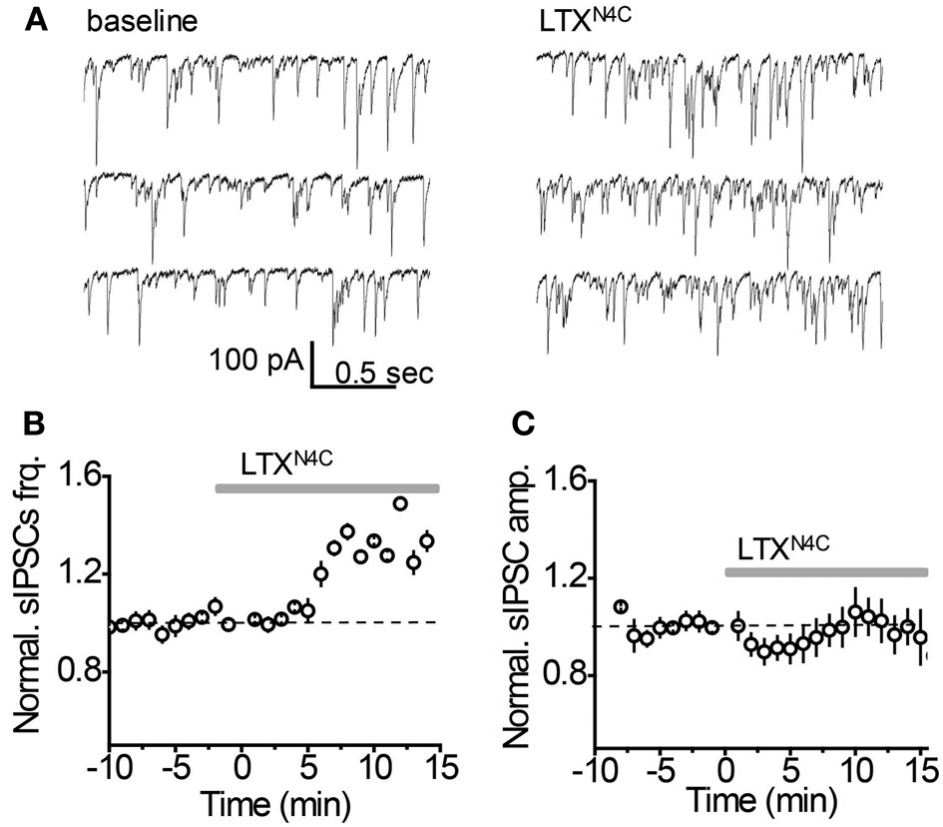
**LTX^N4C^ enhances spontaneous synaptic GABA release. (A)** Representative whole-cell recordings of sIPSC from an individual CA1 *str. radiatum* interneuron before (*left*) and 10 min after (*right*) focal application of LTX^N4C^. **(B)** LTX^N4C^ effect on the mean sIPSC frequency normalized to the baseline *(dashed line*, *n* = 7). *Gray line*—application of LTX^N4C^. (**C**) LTX^N4C^ effect on mean sIPSC amplitude normalized to the baseline *(dashed line*, *n* = 7). *Gray line*—application of LTX^N4C^.

**Figure 6:**
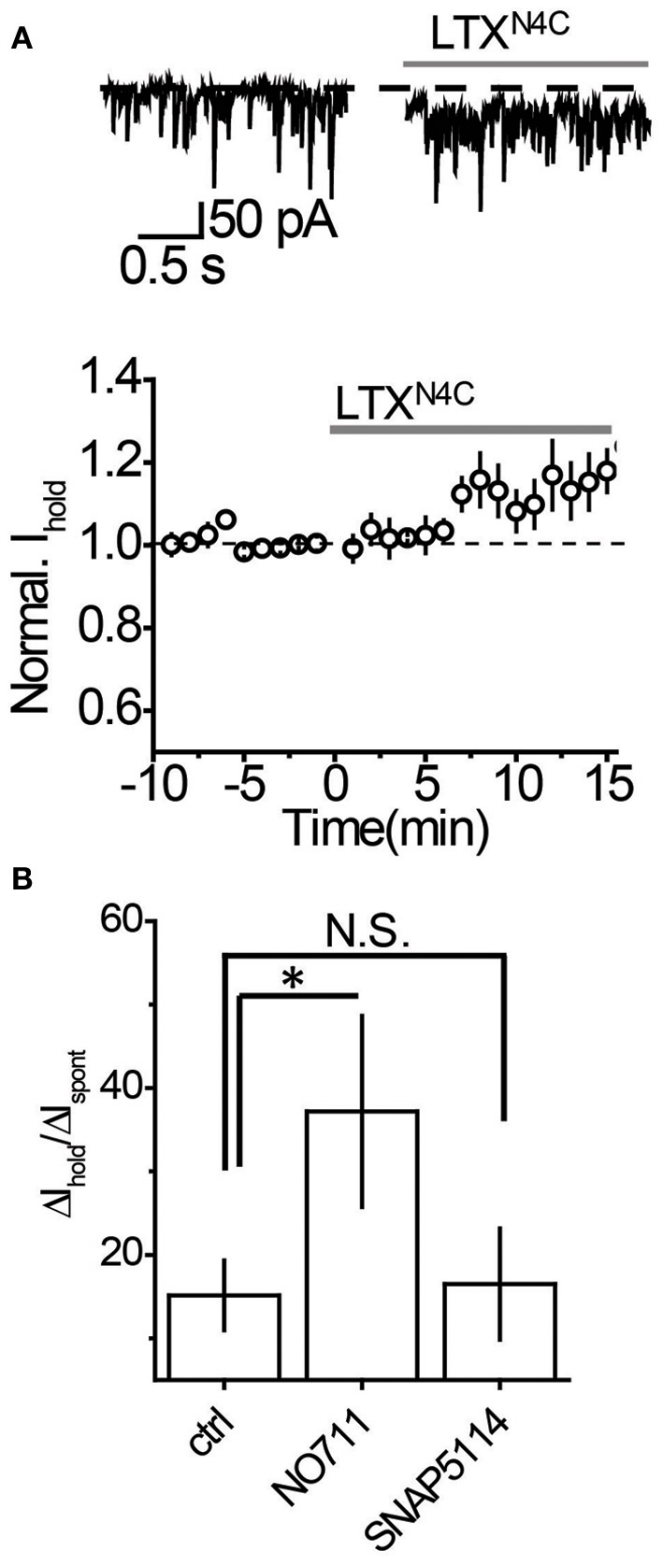
**Tonic GABA_A_ current produced by an increase in vesicular GABA release is regulated by mGAT1, but not mGAT3/4. (A)** *Top*: Representative traces showing an increase in *I*_hold_ 10 min after LTX^N4C^ (*gray line*). *Bottom*: Time-course of normalized *I*_hold_ before and during LTX^N4C^ application (*n* = 7). *Dashed line*—baseline *I*_hold_, *gray line*—application of LTX^N4C^. **(B)** Summary graphs showing an increase in tonic GABA_A_ current with increasing synaptic release (Δ*I*_hold_/Δ*I*_spont_). The increase in the Δ*I*_hold_/Δ*I*_spont_ ratio during NO711 (*n* = 6) application suggests that more GABA escapes the synapses when mGAT1 is blocked. SNAP5114 (*n* = 7) had no effect on the Δ*I*_hold_/Δ*I*_spont_ ratio, suggesting that mGAT3/4 does not regulate GABA escape from the synapses. ^*^P < 0.05, N.S. *P* > 0.05, unpaired *t*-test.

**Figure 7:**
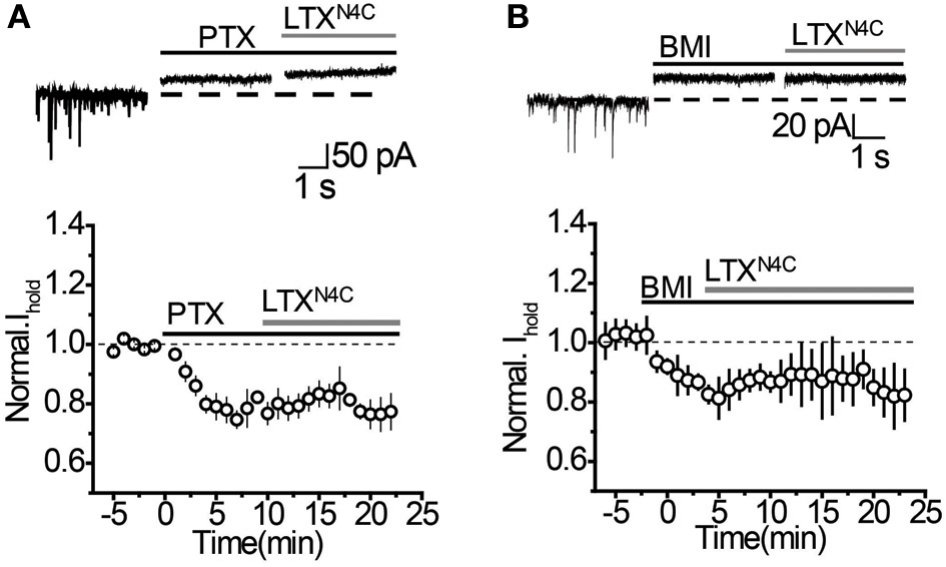
**LTX^N4C^-mediated increase in *I*_hold_ is blocked by GABA_A_ receptor antagonists. (A)** *Top*: Representative traces showing that picrotoxin (PTX, *black solid line*) completely blocks the increase in *I*_hold_ produced by LTX^N4C^ (*gray line*). *Dashed line*—baseline *I*_hold_. *Bottom*: Effect of PTX and LTX^N4C^ on *I*_hold_ normalized to the baseline *(dashed line*, *n* = 4). *Solid black line*—application of PTX, *solid gray line*—application of LTX^N4C^. **(B)** *Top*: Representative traces showing no increase in *I*_hold_ by LTX^N4C^ (*gray line*) in the presence of bicuculline (BMI, *black solid line*). *Dashed line*—baseline *I*_hold_.*Bottom*: Effect of BMI and LTX^N4C^ on *I*_hold_ normalized to the baseline *(dashed line*, *n* = 4). *Solid black line*—application of BMI, *solid gray line*—application.

It remains possible, however, that when vesicular release is increased, mGAT3/4 can contribute to the uptake of GABA escaping from the synaptic cleft together with mGAT1. To test this possibility, synaptic GABA release was enhanced with the mutant α-latrotoxin (LTX^N4C^) which, unlike the wild-type toxin, does not form pores in the membrane and increases neurotransmitter release by stimulating LTX receptors (most likely latrophilin 1) without neuronal depolarization (Ichtchenko et al., 1998; Capogna et al., 2003; Volynski et al., 2003; Deak et al., 2009). LTX^N4C^ at a concentration of 0.1 nM increased the frequency of sIPSCs to 144.5 ± 10.5% of baseline (*n* = 7, *P* = 0.004 paired *t*-test; Figures 5A,B). As expected for LTX^N4C^, which activates latrophilin after a lag-period (Volynski et al., 2004), the observed increase started 5 min after drug application. No significant change in the sIPSC amplitude was detected (107.6 ± 7.6% of baseline, *n* = 7, *P* = 0.178 paired *t*-test; Figure 5C), indicating that LTX^N4C^ did not affect the membrane permeability of the post-synaptic cell. We next tested how changes in vesicular GABA release affect the tonic GABA_A_ current. LTX^N4C^ increased *I*_hold_ in the recorded cells to 117.9 ± 5.1% of baseline (*n* = 6, *P* = 0.007 paired *t*-test; Figure 6A). The time-course of this increase followed the time-course of the change in sIPSC frequency (Figure 6A, compare to Figure 5B). The LTX^N4C^ effect on *I*_hold_ was blocked by picrotoxin (99.58 ± 7.27% of *I*_hold_ in picrotoxin, *n* = 4, *P* = 0.29 paired *t*-test) or by bicuculline (100.9 ± 3.6 % of *I*_hold_ in bicuculline, *n* = 4, *P* = 0.48 paired *t*-test), indicating that the observed change in *I*_hold_ was mediated by GABA_A_ receptors (Figure 7). To estimate the proportion of the tonic GABA_A_ current produced by enhanced vesicular GABA release, we calculated the ratio between the change in *I*_hold_ and the change in *I*_spont_ [Δ*I*_hold_/Δ*I*_spont_, where *I*_spont_ is the time-averaged current mediated by sIPSC calculated as the product of the mean charge transfer of sIPSCs and their frequency (Semyanov et al., 2003; Song et al., 2011)]. In control conditions this ratio was 15.14 ± 4 (*n* = 8), suggesting that *I*_hold_ changes by approximately 15 pA per 1 pA change in *I*_spont_. When GABA uptake is blocked, an increase in Δ*I*_hold_/Δ*I*_spont_ ratio would be expected, indicating that more GABA can escape the synaptic cleft. Since IPSC shape (and thus *I*_spont_) can change in the presence of GABA uptake blockers, we added the uptake blocker first and then measured the effect of LTX^N4C^. The Δ*I*_hold_/Δ*I*_spont_ ratio was increased approximately two-fold in the presence of NO711 (37.20 ± 11.45, *n* = 7, *P* = 0.04 for difference from control, unpaired *t*-test), but was not significantly affected in the presence of SNAP5114 (16.48 ± 6.67, *n* = 5, *P* = 0.11 for difference from control, unpaired *t*-test), suggesting that mGAT1, but not mGAT3/4, is responsible for limiting GABA spillover, even with increased levels of synaptic release (Figure 6B).

## Discussion

Our results demonstrate that mGAT1 and mGAT3/4 transporter types are associated with two different extrasynaptic signaling pathways. mGAT1, but not mGAT3/4, limits synaptic GABA spillover. In contrast, mGAT3/4 makes a larger contribution than mGAT1 to the regulation of GABA released from extrasynaptic sources (Zilberter et al., 1999; Kozlov et al., 2006; Lee et al., 2010).

The different roles of mGAT1 and mGAT3/4 in the regulation of extrasynaptic GABA sources can be explained by their cellular localization. In rat hippocampus, rGAT1 is expressed predominantly in neurons, which express no or only low levels of rGAT3 (Ribak et al., 1996; Heja et al., 2009). In contrast, rGAT3 is strongly expressed in rat hippocampal astrocytes (Heja et al., 2012; Shigetomi et al., 2012). The cell-type specific localization of mGAT1 and mGAT3/4 is responsible for the different regulation of IPSCs by these transporter types. rGAT1 blocker, but not rGAT2/3 blocker, prolongs the decay time of evoked IPSCs in rat neocortex (Keros and Hablitz, 2005) and striatal output neurons (Kirmse et al., 2009). In contrast, in rat *globus pallidus*, both rGAT1 and rGAT2/3 blockers affect the kinetics of evoked IPSCs (Jin et al., 2011). The interpretation of the transporter blocker effects on synaptic signaling is complicated by possible changes in the presynaptic release probability or in the presynaptic excitability due to elevated ambient GABA concentrations.

Notably, expression patterns of the two transporter types can change independently during physiologic processes, such as memory formation (Tellez et al., 2012), and pathologic processes, such as epilepsy (Cope et al., 2009). In addition to transporter expression, the two GABA uptake systems can be individually regulated by endogenous modulators. For example, zinc, a potent inhibitor of rGAT4, can be released in the hippocampus together with glutamate (Cohen-Kfir et al., 2005). Indeed, zinc increases the extracellular GABA concentration in the hippocampus (Takeda et al., 2004). In addition, glutamate uptake by astrocytes increases the intracellular sodium concentration in these cells (Kirischuk et al., 2012). Increased sodium, in turn, decreases the efficiency of (or even reverses) sodium-dependent GABA uptake by astrocytic rGAT2/3 transporters in the rat (Heja et al., 2012). Such uptake modulation of ambient GABA released from non-vesicular sources would allow for high levels of glutamatergic synaptic excitation to increase tonic GABA_A_ conductance in the hippocampus without affecting synaptic GABAergic signaling.

Taken together with our findings, these considerations suggest the existence of two functionally distinct extrasynaptic GABA signaling systems that possess independently regulated uptake machinery. One system is directly operated by GABA escape from the GABAergic synapses and is associated with mGAT1. This system provides direct feedback regulation of GABAergic synaptic activity through regulating the tonic GABA_A_ conductance. The second system is operated by other neurotransmitters that can promote non-vesicular GABA release (e.g., by astrocytes), and is regulated by mGAT3/4. This system represents an indirect network feedback (via non-vesicular GABA release), which also results in changes in tonic GABA_A_ conductance. Notably, when one of the transporter types is blocked the second type compensates for its loss and takes up GABA originating from both sources. Therefore, when both transporters are blocked their effect on tonic GABA_A_ conductance is supra-additive. This opens up the possibility that in cases when GABA release from the vesicular or non-vesicular source overwhelms their “dedicated” transporter type, the other transporter type can assist in GABA clearance. However, we did not find that a moderate increase in vesicular GABA release can overwhelm mGAT1, but it could potentially happen with further increases in GABA release, or in situations when there is a downregulation of mGAT1.

Importantly, we performed the experiments with GABA_B_ receptors blocked. Activation of these receptors may also depend on the source of extracellular GABA. Indeed, different roles played by rGAT1 and rGAT3 in regulating GABA_B_ receptor activation have been shown in thalamus (Beenhakker and Huguenard, 2010). Thus two GABA sources and two types of GABA transporters can form a specific agonist “template” for extrasynaptic GABA_A_ and GABA_B_ receptor activation (Semyanov, 2008).

Another important question is the identity of the recorded interneurons. We identified these cells solely by their location in CA1 *str.radiatum* and non-pyramidal shape, and did not differentiate different interneuronal subtypes (Klausberger and Somogyi, 2008). It is possible that distinct interneurons may express tonic GABA_A_ currents to differing extents as has been shown in neocortex (Vardya et al., 2008). Potentially, the contribution of vesicular and non-vesicular sources of GABA may also differ among different interneuron subtypes and so tonic GABA_A_ conductances could be regulated differently by the two transporter systems. This question is an important subject for future functional studies, especially those which aim to apply our finding to the identification of novel drug targets. Indeed, the functional difference between mGAT1 and mGAT3/4 prompts a refinement of therapeutic targets for drugs acting on the GABA uptake systems. For example, the anti-epileptic drug, tiagabine, a mGAT1 blocker, increases GABA spillover, and can affect the time course of IPSCs (Walker and Kullmann, 2012). Because IPSCs are involved in synaptic computations, tiagabine can potentially have unwanted effects on synaptic network operation. A selective mGAT3/4 transporter blocker should increase extracellular GABA levels without significantly affecting synaptic communication and perhaps lack the side effects characteristic of tiagabine.

## Author contributions

Inseon Song: collection, analysis, and interpretation of most of the data, conception and design of the experiments; Kirill Volynski: preliminary experiments with latrotoxin, interpretation of data; Tanja Brenner: collection and analysis of some supplementary data; Yuri Ushkaryov: production of mutated latrotoxin; Matthew Walker: data interpretation; Alexey Semyanov: project conception, experimental design, data analysis, and data interpretation; All authors discussed the results, contributed to the writing of the article and have approved its final version. All experiments except production of mutated latrotoxin production were performed in RIKEN BSI, Japan.

### Conflict of interest statement

The authors declare that the research was conducted in the absence of any commercial or financial relationships that could be construed as a potential conflict of interest.
